# Effects of Fermented Liquid Feed on Growth Performance, Meat Quality, and Intestinal Microbiota of Yuedong Black Pigs

**DOI:** 10.3390/ani15182657

**Published:** 2025-09-10

**Authors:** Guoqing Han, Shuai Liu, Chunxiu Zhao, Lei Lei, Ran Yi, Zewei Ma, Jinhao Liu, Canjun Zhu, Songbo Wang, Lina Wang, Gang Shu, Qingyan Jiang, Ruifan Wu

**Affiliations:** 1 Guangdong Provincial Key Laboratory of Animal Nutrition Control, National Engineering Research Center for Breeding Swine Industry, State Key Laboratory of Swine and Poultry Breeding Industry, College of Animal Science, South China Agricultural University, Guangzhou 510642, China; hanguoqing020@163.com (G.H.); 15027016245@163.com (S.L.); 13826090723@163.com (L.L.); lcdm-c@outlook.com (R.Y.); vincenttoyyer@163.com (Z.M.); 13553489225@163.com (J.L.); canjunzhu@scau.edu.cn (C.Z.); songbowang@scau.edu.cn (S.W.); wanglina@scau.edu.cn (L.W.); shugang@scau.edu.cn (G.S.); 2 Foshan Huayang Animal Nutrition Products Co., Ltd., Foshan 528000, China; zhaochunxiu2025@163.com

**Keywords:** antioxidant, gut health, meat colors, meat composition, pig native breeds

## Abstract

Fermented liquid feed (FLF) is a microbiologically fermented, highly nutritious and easily digestible feed. This study investigated the effects of FLF on growth performance, carcass traits, meat quality, antioxidant capacity, and intestinal microbes of Yuedong black pigs. The pigs were divided into a control group and FLF group. Compared with the control, FLF increased (*p* < 0.05) the ADG and body weight of Yuedong black pigs. FLF increased (*p* < 0.05) the loin muscle area, and reduced cooking loss and meat color brightness (L*) of longissimus thoracis. Furthermore, FLF reduced (*p* < 0.05) the contents of bitter amino acids, including histidine and arginine. The expression of *MYH4* (a fast glycolytic fiber gene) and *TNNI1* (an inhibitor of muscle contraction) was increased (*p* < 0.05) in longissimus thoracis from the fermentation group. FLF increased (*p* < 0.05) the lipogenesis-related gene expression of *FABP4*, *CEBPα,* and *PPARγ* and the protein level of FATP4. Moreover, FLF increased (*p* < 0.05) serum T-AOC and GPx activity. In addition, FLF improved colonic microbial diversity and increased (*p* < 0.05) the abundance of Fibrobacterota (Phylum)-degrading cellulose and Lachnospiraceae-AC2044-group (genus)-generating butyric acid. In conclusion, FLF has a broad perspective for improving growth performance, meat quality, antioxidant capacity, and intestinal microbiota composition of Yuedong black pigs.

## Simple Summary

Yuedong black pigs are becoming popular in the market for their unique flavor and good quality of meat. However, their growth rate and feed conversion rate are low compared with traditional commercial breeds. Fermented feed has the potential to improve animal growth. This study showed that fermented liquid feed could improve the growth rate of Yuedong black pigs. In addition, fermented liquid feed can improve meat quality of longissimus thoracis by reducing cooking loss and meat color brightness. Fermented liquid feed also improves intestinal microbial diversity and increases the abundance of beneficial bacteria in Yuedong black pigs. In conclusion, this study provides a theoretical basis for improving the growth rate and meat quality of Yuedong black pigs.

## 1. Introduction

With the development of the economy and the gradual improvement of living standards, the peoples’ demand for pork has changed from the demand for quantity to high quality. The Yuedong black pig, a native breed primarily distributed in Guangdong Province, China, and its unique meat quality, is popular on the market. However, Yuedong black pigs show poorer feed conversion ratio and slower growth compared to commercial crossbred pigs. Therefore, it is critical to improve the growth performance to enhance the market competitiveness and sustainable development of indigenous pig breeds.

Fermented liquid feed (FLF) is a new feed process in which feed or feed ingredients are mixed with micro-organisms and fermented by adding water or other liquids [1]. FLF can improve feed conversion efficiency and provide specific benefits to the animal body [2]. In addition to the microbes added by fermentation, feed and feed ingredients may also have other beneficial microbes produced during the fermentation process, which helps to maintain animal gut health and enhance the animal’s body immunity [3,4]. In addition, during the process of feed fermentation, microbes can digest antinutritional factors in feed (phytates, glycinin, beta-conglycinin, and trypsin inhibitor activity) and promote the absorption of nutrients [5,6]. In summary, FLF has a high potential as a new feed for improving animal productivity and health. However, the effects of FLF on Yuedong black pigs remain unclear.

Therefore, this study aimed to investigate the effects of FLF on the growth performance, carcass traits, meat quality, antioxidant capacity, and intestinal microbiota of Yuedong black pigs. These results could provide a theoretical basis for the improvement of growth performance and high-quality pork production of Yuedong black pigs.

## 2. Materials and Methods

### 2.1. Animals and Treatments

A total of 100 healthy 90-day-old Yuedong black pigs (33.79 ± 1.14 kg, castrated male pigs) were randomly selected and divided into 2 treatment groups, each with 5 pens and 10 pigs in each pen. The experiment lasted 105 days. The control group was fed a corn-soybean meal-based diet and the experimental group was fed an FLF. Fermentation of the base diet uses a mixed bacteria pack (*Bacillus subtilis* ≥ 1 × 10^8^ CFU/g, *Enterococcus faecalis* ≥ 1 × 10^8^ CFU/g, *Saccharomyces cerevisiae* ≥ 1 × 10^8^ CFU/g, and *Clostridium butyricum* ≥ 1 × 10^8^ CFU/g). The preparation method for fermented liquid feed was as follows. After mixing the strain package with the basic feed at a mass ratio of 1:50, mix thoroughly at a water-to-feed ratio of 2.5:1 and ferment at room temperature for 6 h. Fermented liquid feed was prepared daily and delivered from the fermentation tank to the feed trough via an automated feeder pipe. The base diet composition and nutrient levels are shown in Supplemental Table S1. Body weights were recorded at the beginning and end of the trial. Feed intake was recorded daily. Feed intake in the fermented liquid feed group was calculated on a dry matter basis. Average daily gain (ADG), average daily feed intake (ADFI), and feed-to-gain ratio (FCR) were calculated. Pigs were fed at 7 a.m. and 3 p.m. every day. The pigs were allowed to feed and drink freely. The experimental barn was a semi-open building with natural ventilation. The barns were washed every day and disinfected regularly to keep the barns dry and clean.

### 2.2. Sample Collection

At the end of the trial, 1 or 2 pigs per pen near the per-pen average weight were selected (6 slaughtered in each group) and transferred in treatment groups to the nearest abattoir for blood collection and slaughter sampling. After blood collection, the supernatant was centrifuged at 3000× *g* for 15 min at 4 °C and stored at −80 °C. Longissimus thoracis (LT) were excised from the 10th–13th ribs on the left side of each carcass to measure meat quality and gene expression analysis. Additional LT samples were collected from the left side of the carcass to measure moisture, intramuscular fat (IMF), and amino acids. After carcass division, a small sample of the LT was taken and fixed using paraformaldehyde, and another sample was placed in liquid nitrogen and frozen, then transferred to a −80 °C refrigerator for storage. The colon contents were collected and stored at −80 °C. A tape measure was used to measure the straight length and the oblique length of the carcass. Skin thickness and backfat thickness were measured using vernier calipers. A pencil was used to trace the outline of the LT on butter paper and measure the area.

### 2.3. Meat Quality Determination

The meat color of LT was measured with a calibrated D-65 light source colorimeter (NR20XE, Sanenshi Technology, Guangzhou, China), and L* (brightness), a* (redness), and b* (yellowness) were recorded (blooming time for 20 min). The measuring aperture of the colorimeter was 20 mm. The light source was D65. The field of view was the CIE 10° standard observer. Before using the colorimeter, it was calibrated with a white calibration plate. The pH value of the LT at 45 min and 24 h after slaughter was measured using a pH meter (Testo-205, Desto, Lenzkirch, Germany). The pH meter was set for automatic temperature compensation according to the manual. LT marbling was assessed by 10 different assessors with reference to a standard scale for pork marbling (NPPC, 1991). Samples of LT strips were weighed and suspended in aerated polyethylene film bags with fish hooks for 24 h at 2–4 °C before being weighed again to determine drip loss. The samples were cut into cubes with sides measuring 2 cm and weighed and recorded as m1. The samples were put into the measuring tube in the refrigerator (4 °C), and were taken out after 24 h. The samples were treated with filter papers to absorb the residual liquid and weighed and recorded as m2. The calculation formula was as follows: Drip loss, % = (m1 − m2)/m1 × 100. The LT was cut into squares with sides of 2 cm and weighed, and were recorded as m1 determination of cooking loss. All samples were then placed in individual self-sealing bags and heated in a water bath at 85 °C for 30 min before being removed and cooled under running water. Finally, the moisture was dried with filter paper and weighed again and recorded as m2. The calculation formula was as follows: Cooking loss rate, % = (m1 − m2)/m1 × 100.

### 2.4. Serum Biochemical Indicators and Antioxidant Capacity

The liver function indices (TP, ALB, ALP, AST, and ALT), renal function indices (CRE, UREA), and glucose and blood lipids (GLU, TG, CHO, HDL, and LDL) were measured by an automatic biochemical analyzer according to the manufacturer’s instructions. Serum Malondialdehyde (MDA, A003-1-2, Nanjing Jiancheng Bioengineering Institute, Nanjing, China), Total antioxidant capacity (T-AOC, A015-2-1, Nanjing Jiancheng Bioengineering Institute, Nanjing, China), and Glutathione peroxidase (GPx, A005-1-2, Nanjing Jiancheng Bioengineering Institute, Nanjing, China) were measured using the relevant kits from Nanjing Jiancheng Bioengineering Institute (Nanjing, China), according to the specific operations as described in the instruction manual.

### 2.5. HE Staining of Muscle, and Determination of IMF

Muscle tissue was cut into 1 cm × 1 cm × 1 cm pieces and fixed in 4% paraformaldehyde. Samples were sent to Wuhan Saiweier Biotechnology Co., Ltd. for staining and preparation. HE images of the LT (section) of Yuedong black pig were analyzed and processed by Image Pro Plus 6.0 (Image Pro Plus, Media Cybernetics, Rockville, MD, USA). Moisture and IMF content in the LT were determined using lyophilization and the Soxhlet extractor method according to the previous methods [7]. Free amino acids were determined according to the previous method [8]. Briefly, freeze-dried samples were mixed with 10% sulfosalicylic acid, centrifuged to remove the supernatant, and, finally, measured using an automated amino acid analyzer (L-8900, Hitachi Ltd., Tokyo, Japan).

### 2.6. RT-PCR (qPCR)

Total RNA was extracted from the LT using the kit (R4130-02, Magen, Guangzhou, China). The total RNA (2 μg) was reverse-transcribed into cDNA with random primers using the M-MLV enzyme (Promega, Madison, WI, USA). qPCR was performed on a 7300HT Fast RT-PCR system (Applied Biosystems, Carlsbad, CA, USA) using specific primers and the 2×SYBR Green master mix, following the manufacturer’s instructions (Q711, Vazyme, Nanjing, China). The sequences of the qPCR primers were shown in Supplemental Table S2. ACTB was a housekeeping gene.

### 2.7. Western Blot

Proteins were extracted from the LT using RIPA lysis buffer (BB-3101-2, BestBio, Nanjing, China). The samples underwent 10% SDS-PAGE electrophoresis and were then transferred to PVDF membrane (Merck Millipore, Darmstadt, Germany). The membrane was blocked in 6% skim milk. The membranes were then placed at 4 °C and incubated with primary antibodies overnight and then incubated with the secondary antibody for 1.5–2 h. The proteins were visualized using Omni-ECL (Epizyme, Shanghai, China). The band intensity was quantified via Image Pro Plus. The primary antibodies used included anti-MYH4 (1:1000, 10F5, DSHB, Iowa City, IA, USA), anti-FATP4 (1:1000, A2640, Proteintech, Rosemont, IL, USA), and anti-GAPDH (1:50,000, P04406, Bioworld, Nanjing, China).

### 2.8. DNA Extraction, 16S rRNA Amplification, and Bioinformatics Analysis

A 16s rRNA sequencing of colon contents was commissioned to Hangzhou Lianchuan Biotechnology Co. Briefly, the total DNA of the contents was extracted and then PCR-amplified using primers for the V3-V4 region. The forward primer sequence was 341F (5′-CCTACGGGNGGCWGCAG-3′). The reverse primer sequence was 805R (5′-GACTACHVGGGTATCTAATCC-3′). AMPure XT beads were used to purify the PCR product. Qubit (Invitrogen, Carlsbad, CA, USA) was used to quantitatively purify the PCR product. Agilent 2100 Bioanalyzer (Agilent, Santa Clara, CA, USA) and Illumina Library Quantification Kit (Kapa Biosciences, Woburn, Massachusetts, USA) were used to analyze amplification products. Sequencing was performed by the NovaSeq PE250 platform.

The samples were sequenced on the Illumina NovaSeq platform. Data cleaning was performed using fqtrim (v 0.94). Data normalization was performed using SILVA (release 132) classifier. Diagrams were implemented using the R package (v3.5.2). Bioinformatics analysis using the Lianchuan biocaloud platform (v1.0).

### 2.9. Statistical Analysis

HE images of the LT tissue (section) of Yuedong black pigs were analyzed and processed by Image Pro Plus 6.0 (Image Pro Plus, Media Cybernetics, Rockville, MD, USA). The Shapiro–Wilk test was used to ensure that the data were normally distributed. For growth performance data, each pen was treated as the experimental unit for statistical analysis. The experimental data, excluding 16S rRNA results, were analyzed using Student’s *t*-test in Graphpad Prism 10.1.2 (GraphPad Software, Inc. San Diego, CA, USA) unless otherwise stated. For bioinformatics analysis of colonic contents, each pig was treated as the experimental unit for statistical analysis. The 16S rRNA results were plotted using the Lianchuan biocaloud platform. The difference between the two groups of microbial data was analyzed using the Wilcoxon rank sum test. Spearman correlation analysis of colonic barrier-related gene levels was conducted with the top 10 differential microbes in genus. Significance was set at *p* < 0.05.

## 3. Results

### 3.1. The Growth Performance of Yuedong Black Pigs

No pigs died or were eliminated during the experiment. The FLF increased (*p* < 0.05) the final weight and ADG of Yuedong black pigs but did not affect (*p* > 0.05) ADFI (dry matter) and FCR (Table 1).

### 3.2. The Carcass Traits of Yuedong Black Pigs

The FLF had no significant effect (*p* > 0.05) on the straight length and carcass oblique length, skin thickness, and average backfat thickness of Yuedong black pigs (Table 2). The FLF significantly increased (*p* < 0.05) the loin muscle (the LT at the 10th rib) area of Yuedong black pigs (Table 2).

### 3.3. The Meat Quality of Yuedong Black Pigs

The FLF had no significant effect (*p* > 0.05) on the pH_45min_ and pH_24h_ of the LT of Yuedong black pigs (Table 3). The FLF reduced (*p* < 0.05) L*, but had no significant effect (*p* > 0.05) on a* and b* (Table 3). There was no significant effect (*p* > 0.05) of the FLF on LT marbling scores, moisture, and IMF content (Table 3). Cooking loss of the LT was significantly lower (*p* < 0.05) in the FLF group (Table 3).

In addition, we also determined the free amino acids in the LT. FLF significantly reduced (*p* < 0.05) the levels of histidine and arginine, two bitter amino acids, suggesting improved pork flavor (Table 4). The proline level was also reduced in the FLF group (Table 4).

### 3.4. The Muscle Development and Fat Deposition of Yuedong Black Pigs

**Figure 1:**
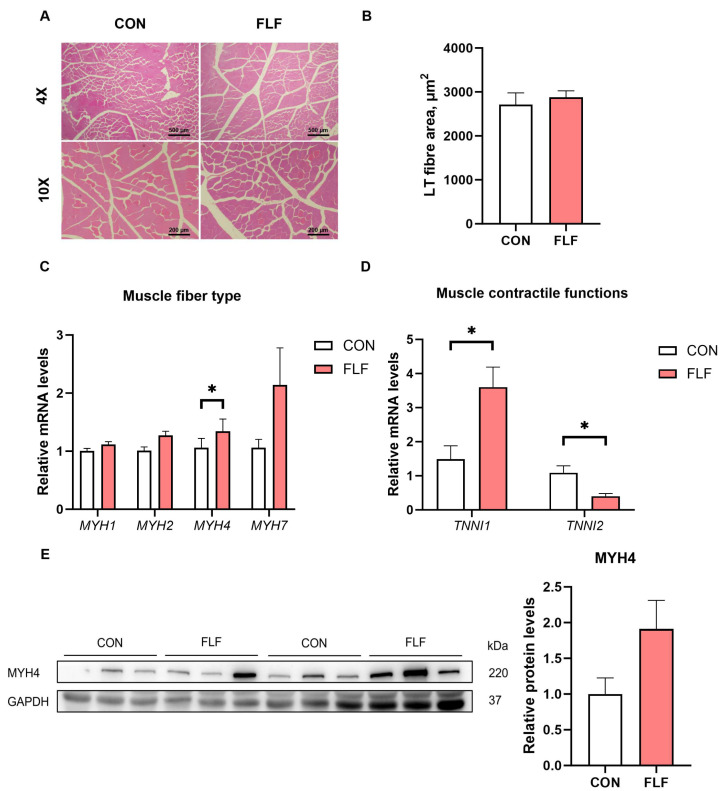
Effects of fermented liquid feed on the muscle fiber of Yuedong black pigs. (**A**,**B**) HE staining and statistical analysis cross-sectional area of longissimus thoracis. (**C**,**D**) Relative mRNA expression of genes related to muscle fiber type and muscle contractile function. (**E**) MYH4 protein levels in the LT muscle were determined by Western blot analysis. *MYH1*, Myosin heavy chain 1; *MYH2*, Myosin heavy chain 2; *MYH4*, Myosin heavy chain 4; *MYH7*, Myosin heavy chain 7; *TNNI1*, Troponin I1; TNNI2, Troponin I2. CON, the control group, was fed basal diet; FLF, fermented liquid feed. The data statistical significance was compared with Student’s *t*-test. Data are presented as the means ± SEM of six independent biological replicates, * *p* < 0.05.

As the FLF increased the loin muscle area, we further determined muscle fiber changes in the LT. The HE-stained sections of the LT showed that FLF did not affect (*p* > 0.05) the muscle fiber area of the LT (Figure 1A,B). Fast glycolytic fiber gene expression of *MYH4* was up-regulated (*p* < 0.05) in the LT of the FLF group, but there was no significant change (*p* > 0.05) in MYH4 protein expression (Figure 1C,E). The FLF had no effect (*p* > 0.05) on the mRNA expression of the *MYH1*, *MYH2*, and *MYH7* genes (Figure 1C). The *TNNI1* (inhibitor of muscle contraction) gene was up-regulated (*p* < 0.05) and the *TNNI2* (promote muscle contraction) gene was down-regulated (*p* < 0.05) in the LT of the FLF group (Figure 1D). The above results suggest that FLF can promote muscle development and reduce muscle contractile function.

**Figure 2:**
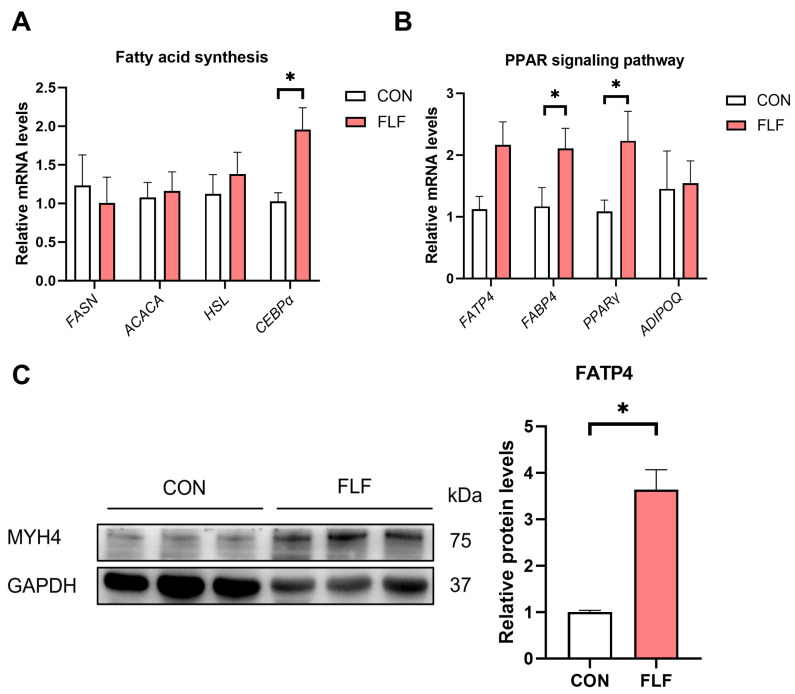
Effects of fermented liquid feed on the genes related to lipid metabolism of Yuedong black pigs. (**A**) Relative mRNA expression of genes related to fatty acid synthesis. (**B**) Relative mRNA expression of PPAR signaling pathway. (**C**) FATP4 protein levels in the LT muscle were determined by Western blot analysis. *FASN*, Fatty acid synthase; *ACACA*, Acetyl-CoA carboxylase alpha; *HSL*, Lipase E; *CEBPα*, CCAAT enhancer binding protein alpha; *FABP4*, Fatty acid binding protein; *PPARγ*, Peroxisome proliferator activated receptor gamma; *ADIPOQ*, Adiponectin. CON, the control group, was fed basal diet; FLF, fermented liquid feed. The data statistical significance was compared with Student’s *t*-test. Data are presented as the means ± SEM of three independent biological replicates, * *p* < 0.05.

The FLF had no effect (*p* > 0.05) on the mRNA expression of *FASN*, *ACACA*, and *HSL* (Figure 2A). The FLF increased (*p* < 0.05) *CEBPα* gene expression in the LT. The expression of *FABP4* and *PPARγ* genes was up-regulated (*p* < 0.05) in the LT of the FLF group (Figure 2B). There were no significant changes in *FATP4* and *ADIPOQ* gene expression (Figure 2 B). However, the FATP4 protein level was significantly up-regulated (*p* < 0.05) in the FLF (Figure 2C). These results suggest that FLF improved lipid metabolism in the LT.

### 3.5. The Serum Biochemical Indicators and Antioxidant Capacity of Yuedong Black Pigs

The FLF had no significant effect (*p* > 0.05) on the liver function indices (TP, ALB, ALP, AST, and ALT) and renal function indices (CRE, UREA) (Table 5). As the FLF improved the growth performance of Yuedong black pigs, we further determined the serum glucose and lipids (Table 5). FLF did not affect (*p* > 0.05) the levels of GLU, TG, CHO, HDL, and LDL (Table 5). In addition, we also measured the antioxidant capacity of serum. The FLF did not affect the serum MDA content of Yuedong black pigs (Table 5). The FLF increased serum T-AOC and GPx activity in Yuedong black pigs. The above results indicated that the FLF could improve the antioxidant capacity of Yuedong black pigs (Table 5).

### 3.6. The Colonic Microbial Composition of Yuedong Black Pigs

**Figure 3:**
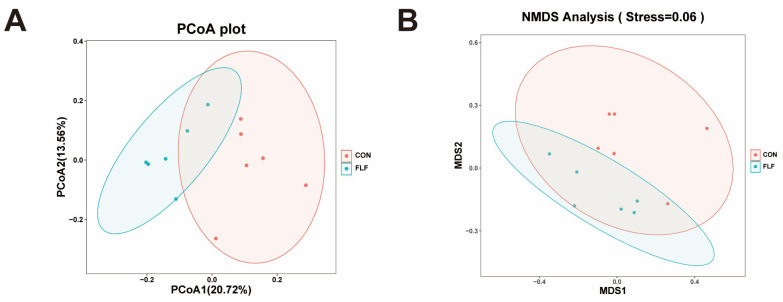
Effects of fermented liquid feed on the colonic microbial beta diversity of Yuedong black pigs. (**A**) Unweighted unifrac distance-based Principal coordinates analysis. (**B**) Bray–Curtis distance-based Nonmetric Multidimensional scaling plot. CON, the control group, was fed basal diet; FLF, fermented liquid feed.

The Chao1 index and Observed OUTs index were significantly up-regulated (*p* < 0.05) in the FLF group, but the Shannon index and Simpson index were unchanged (*p* > 0.05) (Table 6). There were differences in beta diversity between the two groups (Figure 3).

**Figure 4:**
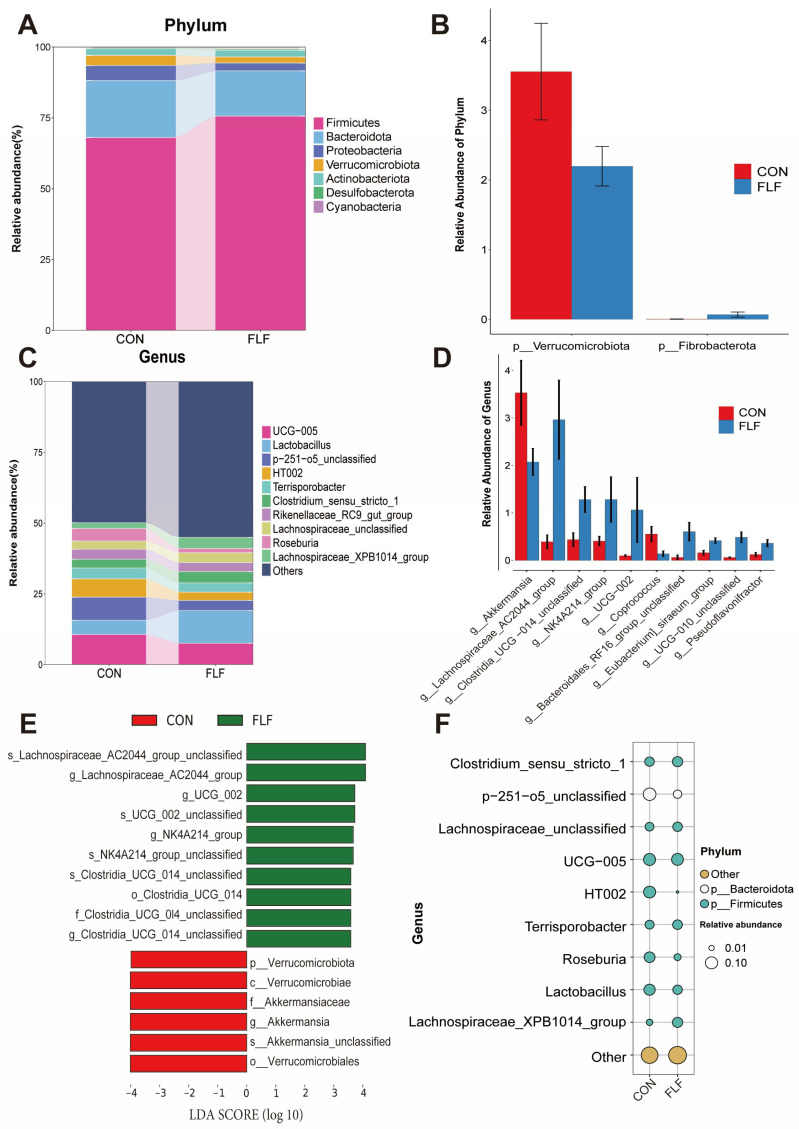
Effects of fermented liquid feed on the colonic microbial species composition of Yuedong black pigs. (**A**,**C**) the stacked bar chart shows the top 10 species in relative abundance at the phylum-level and genus-level. (**B**,**D**) the top 10 species with the highest abundance were screened for *p*-values less than 0.05 and plotted on a bar graph. The data statistical significance was compared with Mann–Whitney U test. (**E**) The LEfSe analysis (LDA score ≥ 3.5) identified the biomarker bacterial species. (**F**) Bubble plots showing genus-level species information and relative abundance and species corresponding to phylum in different groups. CON, the control group, was fed basal diet; FLF, fermented liquid feed.

At phylum level, Firmicutes (phylum, 64.73–70.57%) and Bacteroidota (phylum, 13.95–26.85%) were major phylum (84.52–91.58%) (Figure 4A). Verrucomicrobiota (phylum) abundance was significantly down-regulated (*p* < 0.05) and Fibrobacterota (phylum) abundance was significantly up-regulated (*p* < 0.05) in the FLF group compared to the control group (Figure 4B). The top 10 species (genus level) in relative abundance accounted for 26.72–63.99% of the total, with higher abundance of UCG-005 (genus), Lactobacillus (genus), *p*-251-o5-unclassified (genus), HT002 (genus), and so on (Figure 4C). The abundance of Akkermansia (genus) and Coprococcus (genus) was down-regulated (*p* < 0.05) in the FLF group (Figure 4D). The abundance of Lachnospiraceae-AC2044-group (genus), Clostridia-UCG-014-unclassified (genus), NK4A214-group (genus), UCG-002 (genus), Bacteroidales-RF16-group-unclassified (genus), Eubacterium-siraeum-group (genus), UCG-010-unclassified (genus), and Pseudoflavonifractor (genus) was significantly up-regulated (*p* < 0.05) in the FLF group as compared to the control group (Figure 4D). The LEfSe (LDA scores ≥ 3.5) results showed that 16 differential bacteria were identified (Figure 4E). Bubble chart results showed significant changes in microbial composition in the FLF group. Bubble plot results also showed that FLF significantly affected microbial composition and abundance (Figure 4F).

### 3.7. Effects of FLF on the Colonic Barrier of Yuedong Black Pigs

**Figure 5:**
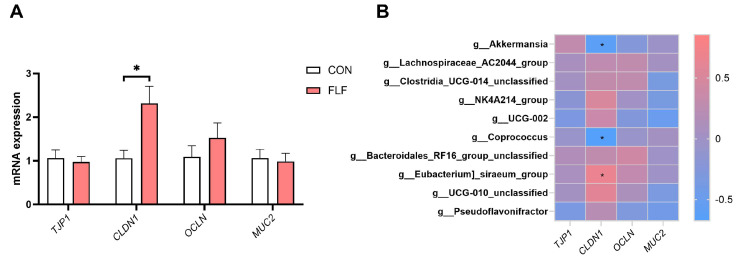
Analysis of changes in the levels of colonic barrier-related genes and their correlation with microbiota. (**A**) Effects of fermented liquid feed on the levels of barrier-related genes. (**B**) Spearman correlation analysis of colonic barrier-related gene levels with the top 10 differential microbes in genus. *TJP1*, tight junction protein 1. *CLDN1*, claudin 1. *OCLN*, occludin. *MUC2*, mucin 2. CON, the control group, was fed basal diet; FLF, fermented liquid feed. Data are presented as the means ± SEM, * *p* < 0.05.

The gut barrier interacts with gut microbes. Since FLF altered the composition of gut microbiota, we measured the levels of colonic barrier-related genes. FLF significantly increased (*p* < 0.05) the gene expression of *CLDN1* genes, but no significant (*p* > 0.05) effect on *TJP1*, *OCLN*, and *MUC2* (Figure 5A). Spearman correlation analysis of colon barrier-related genes with the top 10 differential microbes in genus showed that g-Akkermansia (genus) and g-Coprococcus (genus) showed a significant (*p* < 0.05) negative correlation with the *CLDN1* gene (Figure 5B). g-Eubacterium-siraeum-group (genus) was significantly (*p* < 0.05) positively correlated with the *CLDN1* gene. The above results indicated that the FLF improved the colonic barrier function of Yuedong black pigs.

## 4. Discussion

The indigenous pig breeds in South China are mainly black- and white-colored, characterized by high meat quality, heat resistance, and rough feed tolerance, but there is a significant gap in growth rate and feed efficiency compared with commercial crossbred pigs [9,10]. Therefore, improving the growth performance and increasing the growth rate play a crucial role in enhancing the market competitiveness of Yuedong black pigs.

Liquid fermentation can convert some of the large molecular proteins into small peptides and free amino acids, thus improving nutrient utilization [5]. A study found that feeding weaned piglets for 82 days with wheat-based FLF significantly increased ADG, ADFI, and feed efficiency [11]. In another study, the use of an FLF to feed pigs (weight 44.8–86.8 kg) can increase final weight and ADG, but there were no changes to ADFI and feed conversion ratio [12]. Xin et al. [13] found that FLF feeding increased ADG and ADFI of commercial crossbred pigs from 8 to 125 kg, but had no significant effect on F/G. We found that FLF feeding increased the ADG of Yuedong black pigs. Although the ADFI was elevated and F/G was reduced, neither of them was different. A meta-analysis showed that fermented feed increased ADG, body weight, and F/G in pigs [14]. However, some studies have shown that FLF has no significant effect or even reduces the growth performance of pigs [15,16]. Thus far, there is no consistent conclusion on the effect of FLF on pig growth performance. Different feedstuffs, fermentation strains, and fermentation temperatures can affect the nutrient content of the fermentation product.

Carcass traits are influenced by a variety of genetic, environmental, and nutrient factors. Nutrients ingested by the organism are supplied preferentially to vital organs and physiological processes, followed by bone and muscle development, and, finally, fat deposition [17]. Therefore, improving the body’s nutrient digestion and utilization is necessary to increase the rate of growth and development. The FLF can increase carcass weight, kill-out, backfat thickness, and muscle depth [18]. Fermented mixed feed could increase loin muscle area [19]. In this study, FLF significantly increased loin muscle area, but had no significant effect on other carcass traits. The improved carcass traits of fermented feeds may be related to their increased feed digestibility, which provides more absorbable nutrients to the organism.

Meat quality mainly includes IMF content, marbling, meat color, and drip loss. Myoglobin is the main protein that determines the meat color [20]. A study found that fermented mixed feeds can increase the value of meat color a* and improve marbling scores [19]. In this study, FLF decreased the meat color brightness value (L*) of Yuedong black pigs. Free amino acid content affects the meat taste and flavor [21]. Tang et al. [22] showed that fermented solid feeds increased glutamate (umami taste) in the LT of finishing pigs. Tian et al. [23] showed that the use of fermented feed reduced the content of threonine (bitter) and proline (sweet and bitter) amino acids in the LT of finishing pigs. Our study found that FLF significantly reduced the levels of histidine (bitter), arginine (bitter), and proline (sweet and bitter) in the LT. This suggests that fermented feeds enhance pork taste and flavor by decreasing the content of bitter amino acids, but the exact mechanism needs to be further explored. When muscles are exposed to air, myoglobin oxygenates to form a bright cherry-red color and gradually turns white with increased exposure to oxygen, affecting the senses [24]. Increasing the antioxidant capacity of the organism slows down the process of oxygenation of the carcass muscles, thereby increasing meat color stability [25]. Fermented mixed feeds improved meat color and increased both serum and muscle SOD and GSH-Px activities [19], which is consistent with the results of this study. Previous studies have reported that dietary supplementation with both Bacillus subtilis and Lactobacillus spp. can improve antioxidant capacity in pigs [26,27]. This may be related to microbial metabolites during fermentation. It has been suggested that drip loss of meat may be positively correlated with protein oxidation claims [28]. In this study, cooking loss of LT was reduced in the FLF group, which may be related to the improvement of the antioxidant capacity of the organism. However, it needs to be further explored.

Muscle protein consists of myofibrillar connective tissue and sarcoplasmic proteins. Myofibrillars are the main proteins that make up skeletal muscle [29]. The main muscle fibers are mainly divided into type I muscle fibers and type II muscle fibers. Type II fibers are thicker than Type I fibers [30]. In this study, muscle HE-staining results did not observe a large difference in muscle fiber area, but *MYH4* gene was upregulated in the FLF group. In addition, our study found that FLF increased loin muscle area. This implies that FLF has a positive effect on muscle protein deposition and fiber development, but the specific molecular mechanisms need to be further investigated. TNNI1 and TNNI2 are subunits that regulate skeletal muscle contractile function [31]. The *TNNI1* gene positively correlates with IMF content [32,33,34]. Our study found that FLF significantly increased *TNNI1* gene expression in the LT. Pork with an IMF content of more than 3% will show better palatability and juiciness [35]. Muscle fiber area is positively correlated with IMF content [36]. Liu et al. [37] showed that feeding fermented mixed feed increased IMF content. In this study, there was no significant difference in IMF content between the FLF group and the control group. CEBPα and PPARγ regulate downstream target genes ACACA and FABP4 and promote fat deposition [38]. *FASN*, *FATP1*, and *FABP3* gene expression positively correlates with IMF deposition [39]. Currently, there is less information on the molecular mechanisms of fermented feeds on the expression of fat deposition-related genes. Liu et al. [37] found that fermented mixed feeds up-regulated *CEBPα* and *PPARγ* gene expression in LT. Our study found that *FABP4*, *CEBPα*, and *PPARγ* were significantly upregulated in the FLF group. In conclusion, the increase in IMF content by liquid fermentation material is associated with the regulation of lipogenesis-related genes.

There is a complex crosstalk between gut microbes-host and diet. The intestinal microbiota is regulated by the host’s diet, while compounds produced by gut microbial metabolism also affect host health [40]. Studies have shown that FLF reduces the number of pathogenic bacteria in pigs, thereby reducing the incidence of clinical disease [41]. In this study, FLF improved the biodiversity of colonic microbes in Yuedong black pigs. Gut microbes in finishing pigs can affect pig health, growth performance, and meat quality. Short-chain fatty acids are metabolites of hindgut micro-organisms, which are important for maintaining intestinal barrier integration [42]. Increasing intestinal short-chain fatty acids improved carcass traits and meat color in pigs [43]. In our study, we found that FLF increased the abundance of butyric acid-producing Lachnospiraceae-AC2044 groups (genus). It was found that Fibrobacterota (Phylum) is the main bacterium that degrades lignin and cellulose in the mammalian gut [44]. It has been shown that Fibrobacterota (Phylum), which is enriched in the intestinal tract of Tibetan pigs, is associated with the synthesis of short-chain fatty acids, lactic acid, essential amino acids, and several B vitamins [45]. This implies that the tolerance of Tibetan pigs in facing the extreme environment of the plateau is related to the enrichment of Fibrobacterota (Phylum). In this study, the abundance of colonic microbes Fibrobacterota (Phylum) was higher in the FLF group. In addition, the FLF increased the colonic barrier gene *CLDN1* expression, which was associated with the altered colony composition by FLF. All in all, FLF had beneficial effects on the intestinal microbes of Yuedong black pigs.

## 5. Conclusions

In conclusion, our results suggested that FLF can increase the average daily weight gain and loin muscle area. FLF can improve meat quality by up-regulating lipogenesis-related genes and reducing cooking loss and meat color brightness (L*). The antioxidant capacity of Yuedong black pigs was also improved by FLF. In addition, FLF improved the intestinal microbiota composition by increasing the abundance of beneficial microbiota and improving the colonic barrier function. Thus, our works showed that FLF exhibited significant prospects for improving the economic traits of Yuedong black pigs. Nevertheless, the molecular mechanisms by which FLF improves growth performance, meat quality, and intestinal microbes, require further investigation.

## Figures and Tables

**Table 1:** Effects of fermented liquid feed on the growth performance of Yuedong black pigs.

Item	Diet		SEM	*p*-Value
	CON	FLF		
Initial weight, kg	33.69	33.89	1.14	0.936
Final weight, kg	78.03 ^b^	85.85 ^a^	2.02	0.044
ADG, kg	0.42 ^b^	0.49 ^a^	0.01	0.004
ADFI, kg	1.81	1.99	0.05	0.109
FCR	4.29	4.02	0.09	0.163

Note: CON, the control group, was fed basal diet; FLF, fermented liquid feed. ADG, average daily gain; ADFI, average daily feed intake; FCR, feed-to-gain ratio. Values are shown as mean. SEM is the standard error of the overall mean. The data statistical significance was compared with Student’s *t*-test. Means with different superscript letters are significantly different (*p* < 0.05).

**Table 2:** Effects of fermented liquid feed on the carcass traits of Yuedong black pigs.

Item	Diet		SEM	*p*-Value
	CON	FLF		
Carcass straight length, cm	85.20	87.92	1.36	0.340
Carcass oblique length, cm	75.67	75.00	1.07	0.772
Skin thickness, mm	4.79	4.44	0.29	0.561
Average backfat thickness, mm	34.84	36.07	1.36	0.674
Loin muscle (LT) area, cm^2^	27.08 ^b^	33.08 ^a^	1.37	0.019

Note: CON, the control group, was fed basal diet; FLF, fermented liquid feed. Values are shown as mean. SEM is the standard error of the overall mean. The data statistical significance was compared with Student’s *t*-test. Means with different superscript letters are significantly different (*p* < 0.05).

**Table 3:** Effects of fermented liquid feed on the meat quality of Yuedong black pigs.

Item	Diet		SEM	*p*-Value
	CON	FLF		
pH_45min_	6.78	6.77	0.03	0.903
pH_24h_	5.86	5.92	0.02	0.237
L*	46.0 ^b^	43.0 ^a^	0.60	0.004
a*	8.90	9.20	0.30	0.691
b*	6.00	6.00	0.20	0.985
Marbling scores	1.42	2.17	0.27	0.178
Cooking loss, %	27.78 ^b^	23.20 ^a^	1.17	0.043
Drip loss, %	5.33	2.72	0.77	0.090
Moisture, %	72.22	72.57	0.33	0.624
Intramuscular fat, %	3.36	3.82	0.40	0.592

Note: CON, the control group, was fed basal diet; FLF, fermented liquid feed. pH_45min_, the pH value of meat 45 min after slaughter; pH_24h_, the pH value of meat 24 h after slaughter; L*, brightness of meat 45 min after slaughter; a*, redness of meat 45 min after slaughter; b*, yellowness of meat 45 min after slaughter. Values are shown as mean. SEM is the standard error of the overall mean. The data statistical significance was compared with Student’s *t*-test. Means with different superscript letters are significantly different (*p* < 0.05).

**Table 4:** Effects of fermented liquid feed on free amino acids in the profile of longissimus thoracis in Yuedong black pigs (mg/100 g muscle based on wet weight).

Item	Diet		SEM	*p*-Value
	CON	FLF		
EAA				
Isoleucine	1.44	1.62	0.06	0.099
Leucine	2.38	2.49	0.07	0.474
Lysine	3.72	3.27	0.24	0.367
Phenylalanine	1.40	1.23	0.07	0.205
Methionine	0.82	0.80	0.04	0.758
Threonine	2.54	2.40	0.08	0.400
Valine	3.14	3.19	0.10	0.814
Histidine	1.26 ^a^	0.79 ^b^	0.09	0.001
NEAA				
Serine	1.93	1.68	0.14	0.407
Aspartic acid	1.61	1.63	0.04	0.807
Glutamic acid	2.02	2.41	0.14	0.191
Glutamine	23.47	19.89	1.81	0.346
Glycine	6.02	7.09	0.37	0.162
Alanine	15.61	14.36	0.53	0.253
Tyrosine	1.70	1.66	0.07	0.771
Cysteine	0.59	0.55	0.03	0.488
Arginine	3.19 ^a^	1.61 ^b^	0.30	0.002
Asparagine	0.87	1.14	0.07	0.075
Proline	2.74 ^a^	1.96 ^b^	0.17	0.019
Total EAA	16.70	15.79	0.36	0.220
Total NEAA	59.78	53.99	2.36	0.240
Total amino acid	76.48	69.77	2.59	0.210
UTAA	3.64	4.04	0.15	0.200
STAA	23.56	23.13	0.70	0.770

Note: CON, the control group, was fed basal diet; FLF, fermented liquid feed. EAA, essential amino acids. NEAA, non-essential amino acids. UTAA, umami taste amino acids, Glutamic acid + Aspartic acid. STAA, sweet taste amino acids, Serine + Glycine + Alanine. Values are shown as mean. SEM is the standard error of the overall mean. The data statistical significance was compared with Student’s *t*-test. Means with different superscript letters are significantly different (*p* < 0.05).

**Table 5:** Effects of fermented liquid feed on the serum biochemical indicators and antioxidant capacity of Yuedong black pigs.

Item	Diet		SEM	*p*-Value
	CON	FLF		
Liver function				
TP, g/L	72.67	73.46	1.54	0.813
ALB, g/L	31.67	31.96	0.54	0.802
ALP, U/L	113.18	115.73	8.35	0.887
AST, U/L	47.91	53.20	2.00	0.200
ALT, U/L	53.563	55.67	2.82	0.728
Renal function				
CRE, μmol/L	105.53	108.71	2.85	0.601
UREA, mmol/L	4.17	4.36	0.27	0.756
Glucose and lipids				
GLU, mmol/L	5.11	4.93	0.13	0.522
TG, mmol/L	0.48	0.40	0.03	0.264
CHO, mmol/L	2.37	2.33	0.06	0.796
HDL, mmol/L	0.85	0.78	0.04	0.322
LDL, mmol/L	0.93	0.88	0.03	0.382
Antioxidant capacity				
MDA, nmol/mL	3.05	2.94	0.10	0.586
T-AOC, mmol/L	0.13 ^a^	0.16 ^b^	0.01	0.002
GPx, U/mL	372.30 ^a^	496.62 ^b^	25.77	0.007

Note: CON, the control group, was fed basal diet; FLF, fermented liquid feed. TP, total protein; ALT, alanine transaminase; AST, aspartate aminotransferase; ALP, alkaline phosphatase; CRE, creatinine; ALB, albumin; UREA, carbamide; GLU, glucose; TG, triglyceride; CHO, cholesterol; HDL, high-density lipoprotein; LDL, low-density lipoprotein; MDA, malondialdehyde; T-AOC, total antioxidant capacity; GPx, glutathione peroxidase. Values are shown as mean. SEM is the standard error of the overall mean. The data statistical significance was compared with Student’s *t*-test. Means with different superscript letters are significantly different (*p* < 0.05).

**Table 6:** Effects of fermented liquid feed on the colonic microbial alpha diversity of Yuedong black pigs.

Item	Diet		SEM	*p*-Value
	CON	FLF		
Chao1 index	920.92 ^a^	1100.96 ^b^	33.76	0.002
Observed OTUs index	920.50 ^a^	1100.83 ^b^	33.77	0.002
Shannon index	7.27	7.68	0.18	0.313
Simpson index	0.98	0.96	0.01	0.820

Note: CON, the control group, was fed basal diet; FLF, fermented liquid feed. The data statistical significance was compared with Wilcoxon rank sum test. Means with different superscript letters are significantly different (*p* < 0.05).

## Data Availability

The datasets used in the current study are available from the corresponding author on reasonable request. The names of the repository/repositories and accession number(s) can be found at the following address: https://www.ncbi.nlm.nih.gov/bioproject/PRJNA1192217 (accessed on 30 November 2024).

## Supplementary Materials

The following supporting information can be downloaded at the following address: https://www.mdpi.com/article/10.3390/ani15182657/s1, Table S1: Composition and nutrient levels of the basal diet; Table S2: Primer sequences used for qPCR.

## Abbreviations

The following abbreviations are used in this manuscript:

FLF	Fermented liquid feed
IMF	Intramuscular fat
ADG	Average daily gain
ADFI	Average daily feed intake
FCR	Feed-to-gain ratio
LT	Longissimus thoracis
TP	Total protein
ALB	Albumin
ALP	Alkaline phosphatase
AST	Aspartate aminotransferase
ALT	Alanine aminotransferase
CRE	Creatinine
UREA	Urea
GLU	Glucose
TG	Triglyceride
CHO	Cholesterol
HDL	High-density lipoprotein cholesterol
LDL	Low-density lipoprotein cholesterol
MDA	Malondialdehyde
T-AOC	Total antioxidant capacity
GPx	Glutathione peroxidase
