# Supplementary material for: Effects of Fermented Liquid Feed on Growth Performance, Meat Quality, and Intestinal Microbiota of Yuedong Black Pigs
Source: Animals (Basel). 2025 Sep 10;15(18):2657. doi: 10.3390/ani15182657 (PMC12466584; doi:10.3390/ani15182657)
Supplement: Supplementary file 1 [file animals-15-02657-s001.zip › animals-3766565-supplementary.pdf]

animals

Table S1. Composition and nutrient levels of the basal diet (%, as dry matter basis).

Ingredient	Ratio (%)	Calculated nutrient level	
**Corn**	71.40	ME (MJ/kg)	13.55
**Soybean**	18.00	CP (%)	15.50
**Wheat bran**	8.00	CF (%)	3.64
**Limestone**	1.25	Ca (%)	0.80
**CaHPO4**	0.50	STTD-P (%)	0.22
**NaCl**	0.35	SID Lys (%)	0.85
**L-Lys**	0.20	SID Met (%)	0.24
**Premix** [1]	0.30	SID Thr (%)	0.51
**Total**	100.00	SID Trp (%)	0.14

Note: STTD, standardized total tract digestible. SID, standardized ileal digestibility.[1] Premix provided per kilogram of diet: vitamin A, 5 000 IU; vitamin D_2, 100 IU; vitamin E, 20 IU; vitamin K, 2.2 mg; vitamin B_{12}, 0.03 mg; riboflavin, 4.0 mg; pantothenic acid, 13.8 mg; niacin, 30 mg; choline chloride, 300 mg; folic acid, 0.7 mg; thiamine, 1.5 mg; pyridoxine, 3 mg; biotin, 0.4 mg; Mn, 20 mg; Fe, 75 mg; Zn, 75 mg; Cu, 5 mg; I, 0.14 mg; Se, 0.3 mg.

Table S2. Primer sequences used for qPCR.

Gene	Forward primer sequence (5′-3′)	Reverse primer sequence (5′-3′)
MYH1	TCTTCTCCATCTCCGACAA	GGTTCCTCCTTCTTCTTCTC
MYH2	TCAGTGTAGCCAGCATTG	CAGGTCAGCAGAGTTCAG
MYH4	TCTTCTCCATCTCCGACAA	GGTTCCTCCTTCTTCTTCTC
MYH7	GCCGTGTATGAGAAGATGT	CCAGCCTATGATGTTGTAGT
TNNI1	AGGTGGAGGTAGTGGATG	CTTCACGGACTTGAGGTT
TNNI2	CGGAGAAGCAGAACTACC	CCTCTTGAACTTGCCTCT
FASN	AGCATCTCAAGCCTATCCT	CCTCCACCACCTTCATCT
ACACA	GTCCTTCTCCTCCAACCT	CCTGTCGTCCTCAATGTC
HSL	TGAAGGACAGGACAGTGA	CAGCAGTAGGCGTAGAAG
CEBPα	AAGCCAAGAAGTCGGTAGA	GGTCATTGTCACTGGTCAG
FATP4	GCTCATCAACACCAACCT	GGAGGCAGTCATAGAGGA
FABP4	AGTCAAGAGCACCATAACC	TTCCACCACCAGTTTATCAT
PPARγ	TGTCTCATAACGCCATCAG	GGTCATTCAGGTCAAGGTT
ADIPOQ	GGTCTACTTGAAGGATGTGA	GGAAGCCTGTGAAGATGG
TJP1	AGCTGTTCCTGTGAGTCCTT	TTTCCTGCCCAATTCCCTCA
CLDN1	CCCGGTCAATGCCAGATATG	GCGAAGGTTTTGGATAGGGC
OCLN	CTGTGTAAGGCCCACACCTC	ATTCTGGTACCCACAGTGCC
MUC2	GGCTGGCTGGATTCTGGTAA	CCTGGCTTCTGGTCTTGGTT
ACTB	GTTCGAGACCTTCAACACCC	TCTCCGGAGTCCATCACGAT

Note: *MYH1*, Myosin heavy chain 1; *MYH2*, Myosin heavy chain 2; *MYH4*, Myosin heavy chain 4; *MYH7*, Myosin heavy chain 7; *TNNI1*, Troponin I1; *TNNI2*, Troponin I2; *FASN*, Fatty acid synthase; *ACACA*, Acetyl-CoA carboxylase alpha; *HSL*, Lipase E; *CEBPα*, CCAAT enhancer binding protein alpha; *FABP4*, Fatty acid binding protein; *PPARγ*, Peroxisome proliferator activated receptor gamma; *ADIPOQ*, Adiponectin; *FATP4*, Solute carrier family 27 member 4; *CLDN1*, Claudin 1; *MUC2*, Mucin 2; *TJP1*, Tight junction protein 1; *OCLN*, Occludin; *ACTB*, Actin beta.
